# Retinal Changes Precede Visual Dysfunction in the Complement Factor H Knockout Mouse

**DOI:** 10.1371/journal.pone.0068616

**Published:** 2013-07-02

**Authors:** Jennifer A. E. Williams, John Greenwood, Stephen E. Moss

**Affiliations:** Department of Cell Biology, UCL Institute of Ophthalmology, London, United Kingdom; Eye Hospital, Charité, Germany

## Abstract

We previously reported that aged mice lacking complement factor H (CFH) exhibit visual defects and structural changes in the retina. However, it is not known whether this phenotype is age-related or is the consequence of disturbed development. To address this question we investigated the effect of *Cfh* gene deletion on the retinal phenotype of young and mid-age mice. *Cfh* ^−/−^ mouse eyes exhibited thickening of the retina and reduced nuclear density, but relatively normal scotopic and photopic electroretinograms. At 12 months there was evidence of subtle astroglial activation in the *Cfh* ^−/−^ eyes, and significant elevation of the complement regulator, decay-accelerating factor (DAF) in Müller cells. In the retinal pigment epithelium (RPE) of young control and *Cfh* ^−/−^ animals mitochondria and melanosomes were oriented basally and apically respectively, whereas the apical positioning of melanosomes was significantly perturbed in the mid-age *Cfh* ^−/−^ RPE. We conclude that deletion of *Cfh* in the mouse leads to defects in the retina that precede any marked loss of visual function, but which become progressively more marked as the animals age. These observations are consistent with a lifelong role for CFH in retinal homeostasis.

## Introduction

Age-related macular degeneration (AMD) is the most common cause of blindness in the western world, yet the underlying molecular pathology is poorly understood [1]. A number of single-nucleotide polymorphisms (SNPs) in genes encoding proteins of the complement system have been linked to increased risk of developing AMD. The first to be described, and the best characterised, is a SNP in complement factor H (CFH) that changes a tyrosine to a histidine at position 402 [2]–[6]. CFH functions as a negative regulator of complement activation, in part by binding to serum proteins such as complement C3, heparin and C-reactive protein [7]. CFH also binds to various products of lipid peroxidation to suppress inflammation and macrophage infiltration into the eye. However, recent investigations have shown that the AMD-associated CFH^402H^ variant exhibits decreased binding to malondialdehyde and oxidized phospholipids and is associated with increased complement activation [8], [9].

In previous work we used mice containing a targeted disruption of the *Cfh* gene to investigate the role of CFH in the retina [10]. The *Cfh* ^−/−^ mice were originally developed as a model of membranoproliferative glomerulonephritis, which manifests in these animals as a consequence of uncontrolled C3 activation [11]. In two-year old *Cfh* ^−/−^ mice, we observed a number of abnormalities including reduced visual acuity and loss of rod function, and structural changes to the photoreceptors and Bruch’s membrane but it is not known when these functional and anatomical abnormalities begin in *Cfh* ^−/−^ mice. In a separate study we performed a microarray analysis of neural retina and retinal pigment epithelium (RPE) from these mice to investigate the consequences of *Cfh* deletion on age-related changes in gene expression, and observed anomalies in the expression patterns of certain complement regulators as a function of age [12].

These studies suggest that CFH has a homeostatic or protective role in the retina, a notion further supported by work showing that suppression of CFH expression in the mouse eye using siRNA leads to a more aggressive response to laser-induced choroidal neovascularisation [13]. Although there are recognised differences between the human and mouse complement systems [14], studies in mice may nevertheless provide insight into the role of CFH in the human retina. Here we analysed the phenotype of the 12 month old *Cfh* ^−/−^ mouse retina to determine whether the abnormalities in two year-old mice represent the culmination of a lifelong deterioration or are an early consequence of disturbed development.

## Results

**Figure 1:**
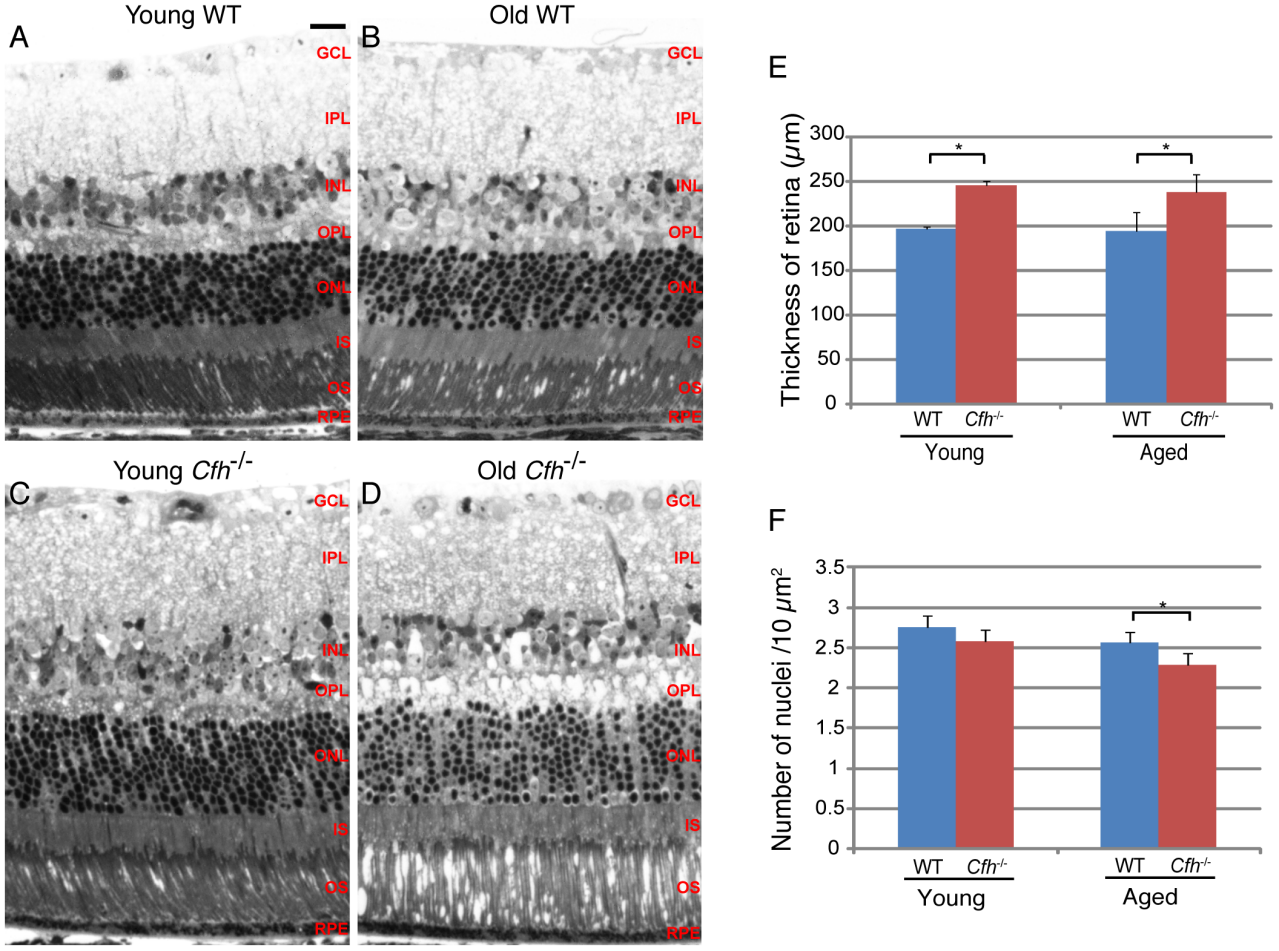
Loss of CFH causes thickening of the retina and reduced nuclear density in the outer nuclear layer. Toluidine blue stained 2 µm semithin sections of (A and B) wild-type and (C and D) *Cfh* ^−/−^ mouse eyes at age 7–8 weeks (A and C) and one year (B and D). Retinal thickness (E) was calculated from the ILM to RPE. Data are means ± S.D., n = 3. Average nuclei number (F) was calculated per 100 µm^2^ area of the ONL. Data are means ± S.D., n = 5. Student t-tests were applied to data, *p = <0.05. Scale bar represents 20 µm.

We first assessed whether the loss of CFH caused any morphological disorganisation in the retinas of young or 1 year *Cfh* ^−/−^ mice. We compared *Cfh* ^−/−^ mice at 7–8 weeks (young) and 1 year (mid-age), and used wild-type age-matched controls to monitor changes associated with normal ageing. Semi-thin resin sections were cut from fixed eyes in all four groups and stained with toluidine blue (Figure 1, A-D). We observed a greater tendency for photoreceptor detachment in the 1 year *Cfh* ^−/−^ samples than in aged-matched wild-type samples and a modest increase in retinal thickness. Quantification of retinal thickness, as measured from the inner limiting membrane (ILM) to the RPE at a position 0.5 mm from the optic nerve head, confirmed a small but significant thickening of the retina in the *Cfh* ^−/−^ mice that was already present at 7–8 weeks and that remained relatively stable up to one year (Figure 1E). Quantification of the nuclei in the outer nuclear layer (ONL) revealed that there was a small reduction in photoreceptor density with age in both wild-type and *Cfh* ^−/−^ mice however this trend did not reach statistical significance (Figure 1F). Whilst wild-type and *Cfh* ^−/−^ nuclear densities were not significantly different at 7–8 weeks, they became so by mid-age, with accelerated photoreceptor cell loss in the *Cfh* ^−/−^ retinas.

**Figure 2:**
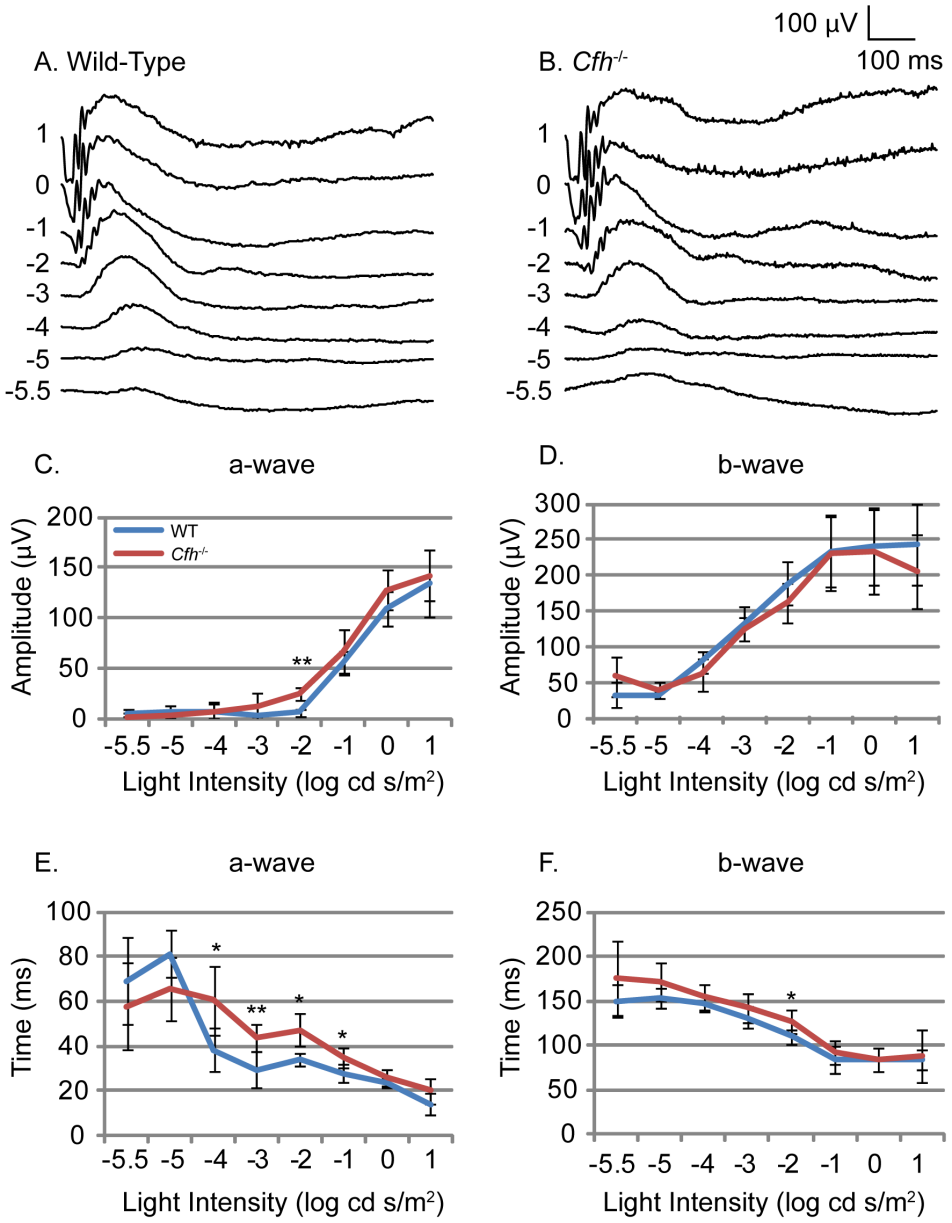
Electroretinogram response to light under scotopic conditions in wild-type and *Cfh* ^−/−^ mice. Electrophysiological assessment of retinal function by electroretinogram (ERG) was performed on one year old *Cfh^−/−^* and wild-type mice. Dark-adapted mice were used to assess scotopic neural retinal responses. Representative ERG traces (A and B) in response to flash stimuli of increasing log intensity. Mean amplitude of a-wave (C) and b-wave (D) measured from wild-type (blue line) and *Cfh* ^−/−^ (red line) mice. Time to peak of the a-wave (E) and b-wave (F) measured from wild-type and *Cfh* ^−/−^ mice. WT n = 6, KO n = 5. Unpaired Student’s t-tests were applied to data, *p<0.05, **p<0.01. ANOVA showed a significant increase in time to peak of a-wave (p<0.01).

In order to assess whether the morphological changes described here had any functional significance we performed ERGs. Dark-adapted 1 year *Cfh* ^−/−^ and age-matched wild-type mice were exposed to flashes of light of increasing intensity. Overlaid scotopic ERG traces from both *Cfh* ^−/−^ and wild-type mice showed clear a- and b- waves (Figure 2, A and B). Retinal function was assessed between groups by comparing the amplitude and time to peak for each wave. The amplitudes of both the a- and b-waves were not significantly different in 1 year *Cfh* ^−/−^ mice compared to wild-type controls, and were similar to values reported in other 1 year pigmented mice (Figure 2, C and D) [15]. However, the time to reach the peak of the a-wave wave was increased significantly in the 1 year *Cfh* ^−/−^ mice compared to wild-type (p = 0.004; Figure 2E) whereas the increased trend in the b-wave did not reach significance (Figure 2F). An unpaired Student t-test for each light intensity revealed that half of the a-wave measurements were significantly increased in *Cfh* ^−/−^ mice.

**Figure 3:**
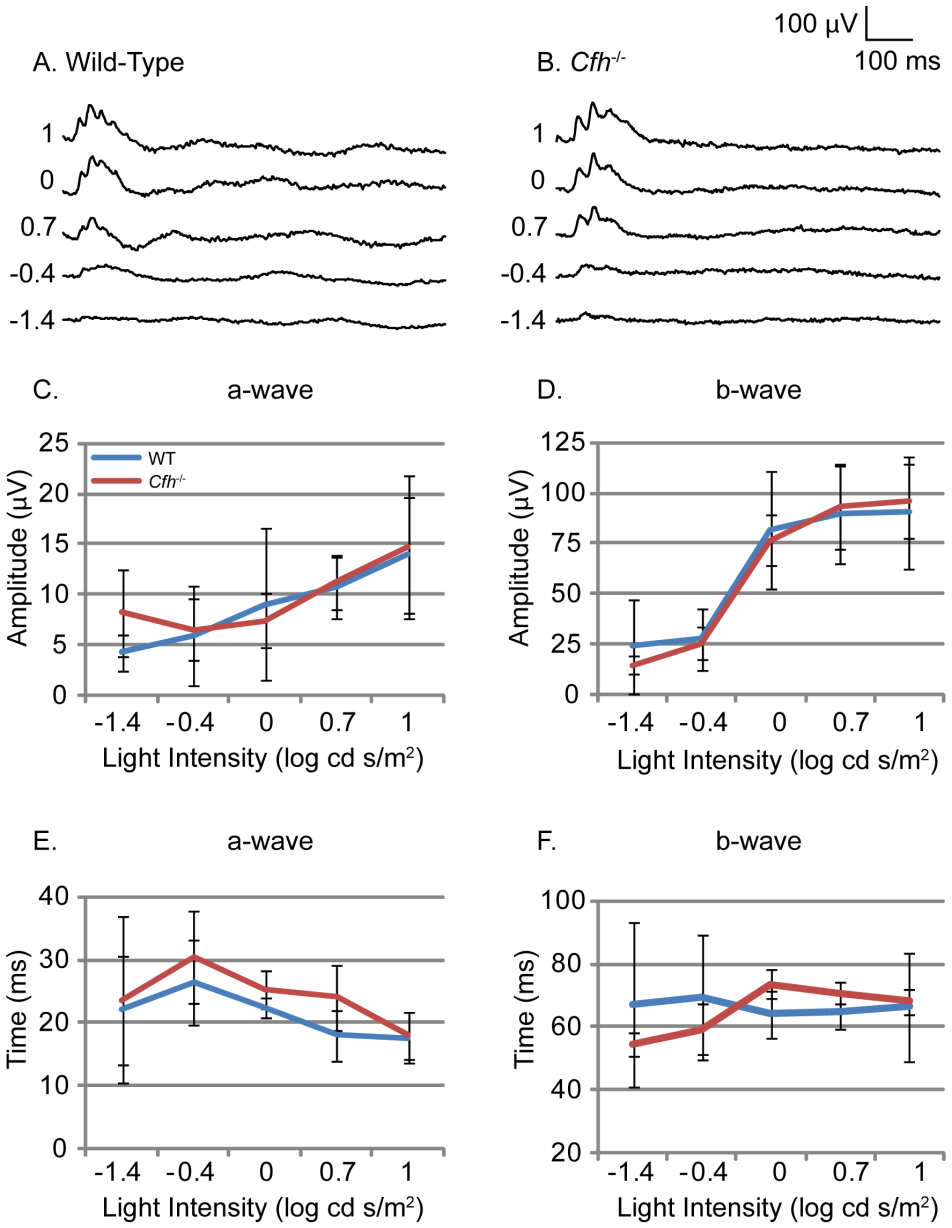
Electroretinogram response to light under photopic conditions in wild-type and *Cfh* ^−/−^ mice. Electrophysiological assessment of retinal function by electroretinogram (ERG) was performed on one year old *Cfh^−/−^* and wild-type mice. Light-adapted mice were used to assess photopic neural retinal responses. Representative ERG traces (A and B) in response to flash stimuli of increasing log intensity. Mean amplitude of a-wave (C) and b-wave (D) measured from wild-type (blue line) and *Cfh* ^−/−^ (red line) mice. Time to peak of the a-wave (E) and b-wave (F) measured from wild-type and *Cfh* ^−/−^ mice. WT n = 6, KO n = 5.

After recording scotopic ERGs, mice were light-adapted for photopic ERGs in order to assess their cone-mediated responses. Overlaid photopic ERG traces from both *Cfh* ^−/−^ and wild-type mice showed small a-wave responses at low light intensities but clear b-wave responses at higher light intensities (Figure 3, A and B). Measurements of the a- and b-wave amplitudes showed no significant difference between 1 year *Cfh* ^−/−^ and age-matched wild-type controls (Figure 3, C and D). There were also no significant differences in the time to peak for both the a- and b-wave responses (Figure 3, E and F). These results suggest that cone-mediated vision is unaffected by the loss of CFH in mice at 1 year of age. VEPs were recorded simultaneously to confirm that neuronal stimulation in the eye elicited subsequent neuronal activity in the visual cortex. VEP recordings from both *Cfh* ^−/−^ and wild-mice showed a negative wave that followed the b-wave of the ERG, indicating normal neural transmission (data not shown). Comparing the amplitude and time to peak for the negative wave of *Cfh* ^−/−^ to wild-type mice did not show any significant differences.

**Figure 4:**
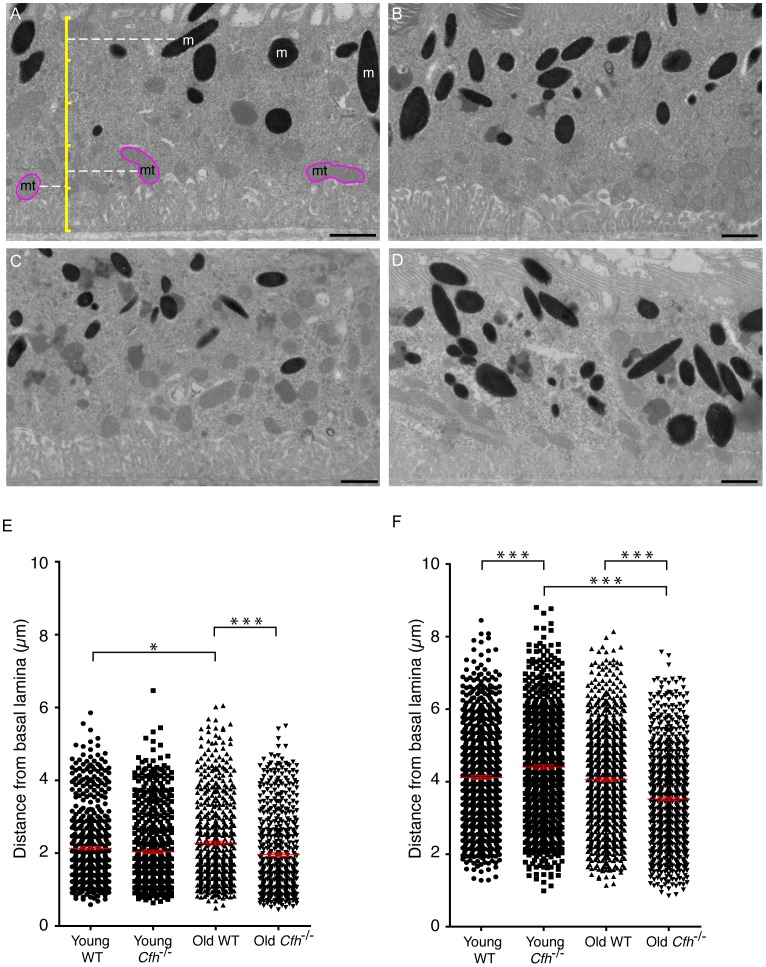
Organelle positioning in the retinal pigment epithelium. Transmission electron micrographs of 70–80 nm sections of RPE from (A & B) 7–8 week old and (C & D) 1 year old (A & C) wild-type and (B & D) *Cfh* ^−/−^ mice. Scale bars represent 1 µm. In (A) an illustrative scale bar (yellow) shows how the positions of melanosomes (m) and mitochondria (mt) were mapped, so that the distances of (E) mitochondria and (F) melanosomes were measured from the basal lamina. Each scatter plot shows quantification of >500 mitochondria or >750 melanosomes per group where each dot represents one organelle. *P<0.05; ***P<0.001. n = 5.

In order to perform detailed ultrastructural examination of the RPE, transmission electron micrographs were prepared from 7–8 week and 1 year *Cfh* ^−/−^ and wild-type mice (Figure 4, A-D). In 2 year wild-type mice, electron-dense material accumulates in the basal region within the RPE but this is decreased in *Cfh* ^−/−^ retinas [10]. In both young and 1 year old samples, electron micrographs showed clear basal infoldings with no sign of any accumulation of electron-dense material towards the basal surface. In both *Cfh* ^−/−^ and wild-type samples there was an expected increase in lipofuscin with age which is consistent with previous studies [16], although levels of lipofuscin did not appear significantly different between genotypes.

In the 2 year *Cfh* ^−/−^ animals melanin and lipofuscin-containing organelles were more evenly distributed throughout the cell compared to age-matched wild-type controls where they were predominantly apical [10]. Here, we analysed the distribution of both mitochondria and melanosomes in the RPE of young and mid-age *Cfh* ^−/−^ and age-matched wild-type mice (Figure 4, E and F). The cross-sectional depth of the RPE cells, measured from the basal lamina to the base of the apical processes, was not significantly different across the four groups with an average height of 5 µm. Mitochondria are normally localised towards the basal side of RPE where there is a large energy demand [17], and the distribution of mitochondria was not significantly affected by the loss of CFH at 7–8 weeks (Figure 4E). However, ageing caused mitochondria in wild-type RPE to become slightly less polarised and they were on average further from the basal lamina which is consistent with published data [18]. In 1 year *Cfh* ^−/−^ samples we observed the opposite whereby mitochondria remained significantly closer to the basal lamina.

Melanosomes are typically polarised towards the apical surface of RPE. Their position is suited to the absorption of scattered light, and thus protection of the basally arranged mitochondria from free radical generation. In wild-type retinas, melanosomes were on average 2 µm further from the basal lamina than mitochondria and their position was not altered with ageing (Figure 4F). However, in the RPE of young *Cfh* ^−/−^ mice, melanosomes were slightly more apical than in wild-type mice. In contrast, in 1 year *Cfh* ^−/−^ retinas, the opposite was observed, in that melanosomes were significantly less polarised. These analyses show that in 1 year *Cfh* ^−/−^ samples the basal polarity of mitochondria was accentuated, whereas there was a significant loss of apical polarity of melanosomes.

**Figure 5:**
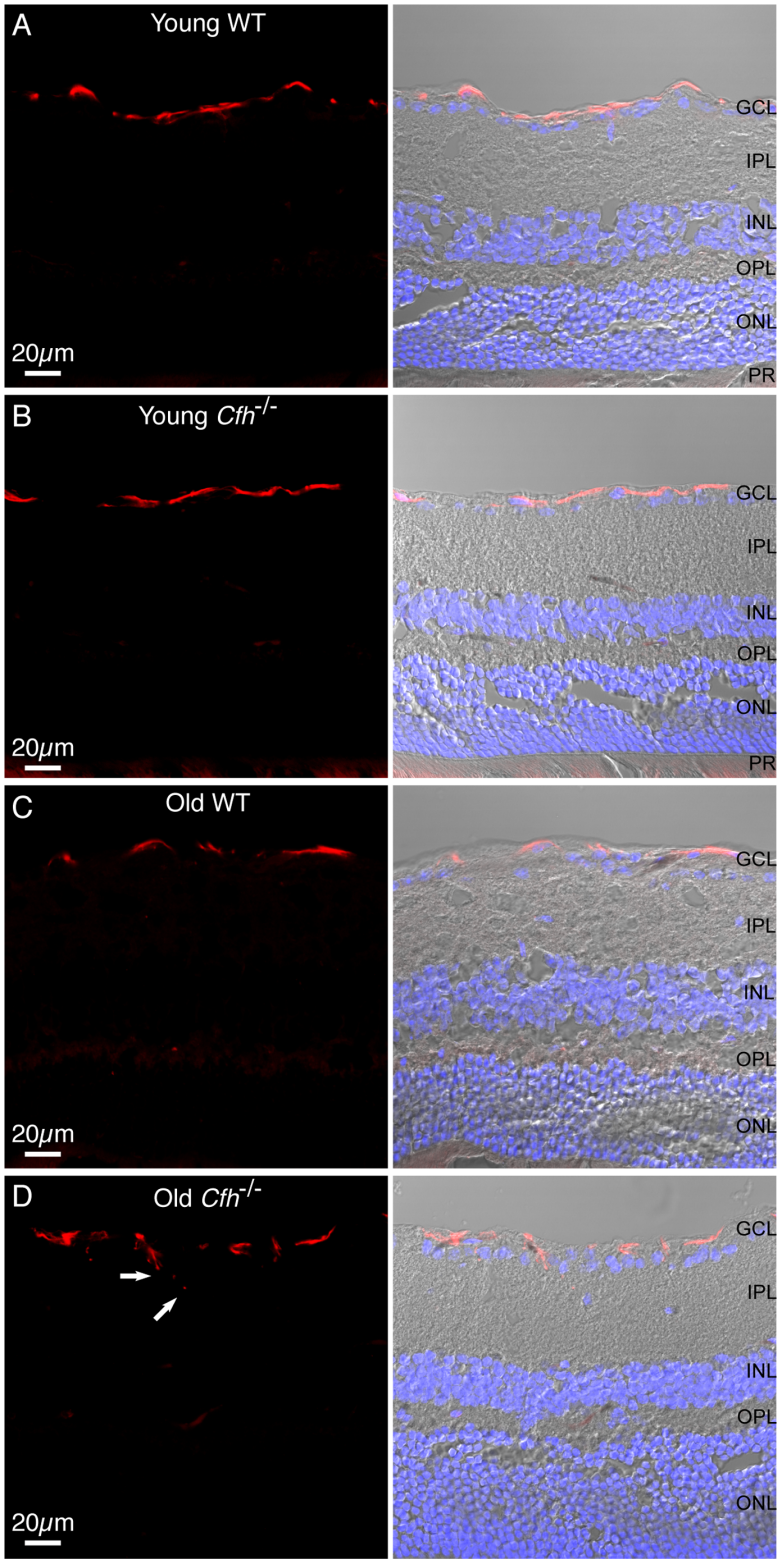
GFAP expression in astrocytic glial cells in *Cfh* ^−/−^ and wild-type mice. 12 µm sections from young (A and B) and one year (C and D) wild-type and *Cfh* ^−/−^ fixed mouse eyed were stained for GFAP (red) and nuclei (blue). Sections were imaged using confocal and differential interference contrast microscopy. Arrows indicate astrocytic processes extending into IPL. GCL, ganglion cell layer; IPL, inner plexiform layer; INL, inner nuclear layer; OPL, outer plexiform layer; ONL, outer nuclear layer; PR, photoreceptors. Images are representative of three independent experiments. Scale bar = 20 µm.

**Figure 6:**
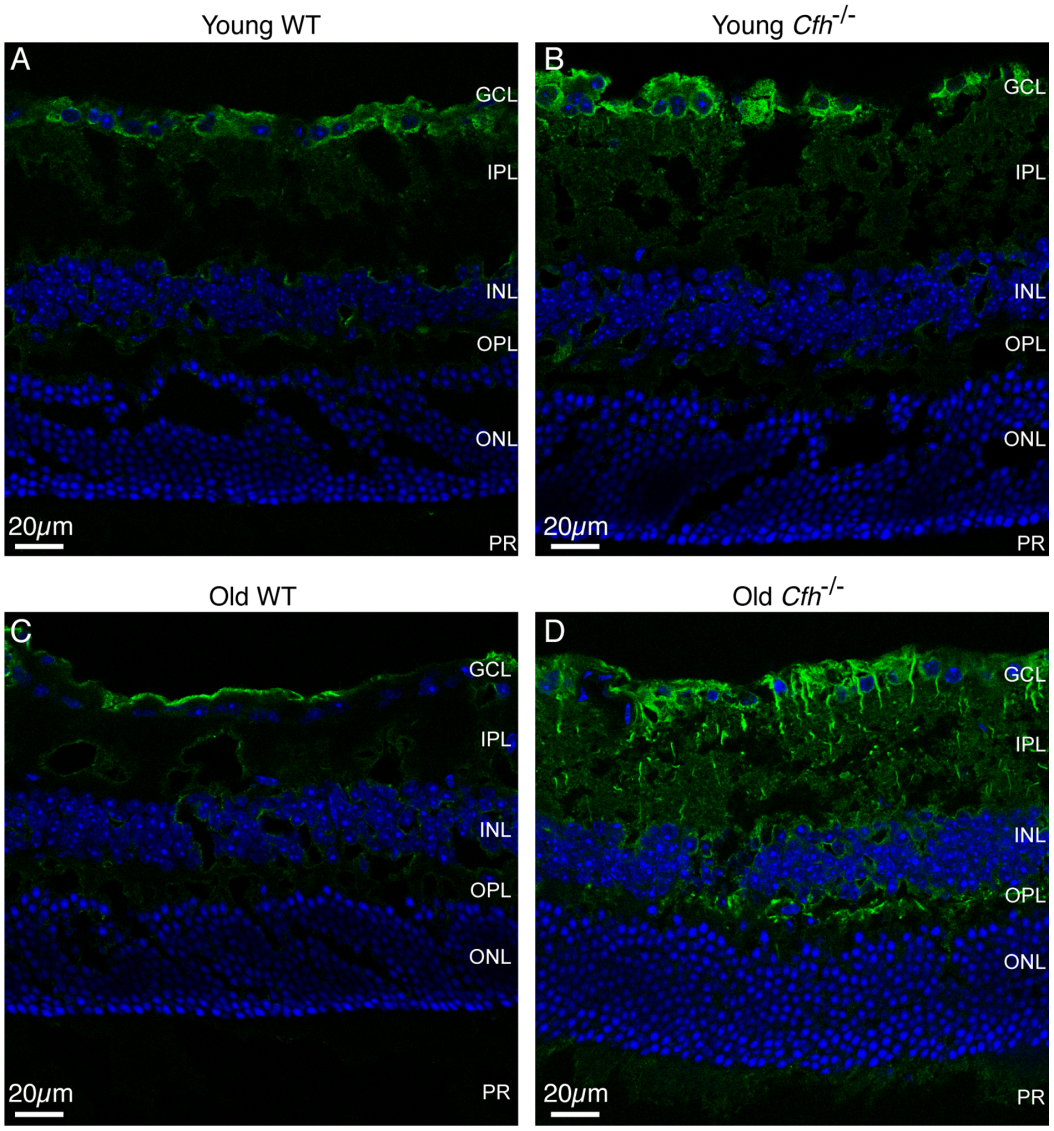
Decay-accelerating factor expression in aged *Cfh* ^−/−^ mice. 12 µm PFA-fixed sections from young (A and B) and old (C and D) wild-type (A and C) and *Cfh* ^−/−^ (B and D) retinas were stained for DAF (green) and nuclei (blue). Sections were imaged using confocal microscopy. GCL, ganglion cell layer; IPL, inner plexiform layer; INL, inner nuclear layer; OPL, outer plexiform layer; ONL, outer nuclear layer; PR, photoreceptors. Images are representative of three independent experiments. Scale bar = 20 µm.

**Figure 7:**
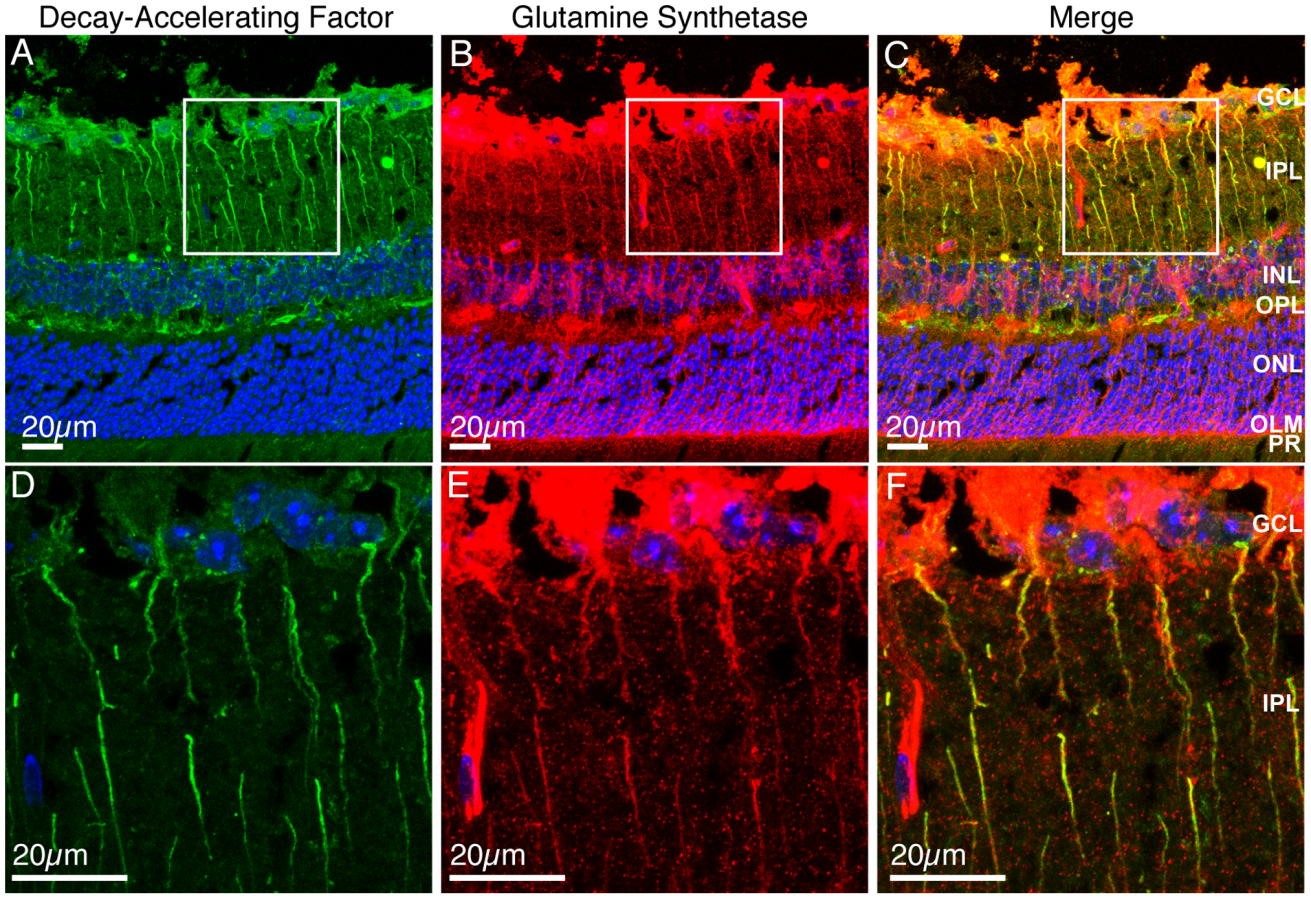
Decay-accelerating factor expression co-localises with glutamine synthetase in Müller cells. 12 µm PFA-fixed sections from old *Cfh* ^−/−^ retinas were stained for DAF (green), glutamine synthetase (red) and nuclei (blue). Maximum intensity projections were made of a 9 µm z-stack of DAF and nuclei (A and D), glutamine synthetase and nuclei (B and E) or merge (C and F). White boxes of low power images (A-C) indicate regions where high power images were taken (D-F). GCL, ganglion cell layer; IPL, inner plexiform layer; INL, inner nuclear layer; OPL, outer plexiform layer; ONL, outer nuclear layer; OLM, outer limiting membrane; PR, photoreceptors. Images are representative of three independent experiments. Scale bars = 20 µm.

Next we examined the expression of glial fibrillary acidic protein (GFAP), a marker of retinal stress. In the wild-type and young *Cfh* ^−/−^ retinas GFAP expression was restricted to astroglia in the ganglion cell layer (GCL) (Figure 5, A and B). However, in 1 year *Cfh* ^−/−^ mice the GFAP positive cell processes were more disorganised than the structures seen in the control retinas and were seen extending towards the inner plexiform layer (IPL) (Figure 5, C and D), providing evidence of astroglial activation in 1 year *Cfh* ^−/−^ retinas. Finally, we investigated the expression of decay-accelerating factor (DAF) which, like CFH, can potentiate the decay of C3 convertases. Staining of DAF in retinal sections revealed positive staining in the GCL in wild-type mice which was unaffected by age (Figure 6, A and B). This expression pattern matched that observed in young *Cfh* ^−/−^ mice (Figure 6C), whereas in the 1 year *Cfh* ^−/−^ retina, DAF-positive projections extended from the GCL into the IPL (Figure 6D). Positive staining was also present amongst the nuclei of the inner nuclear layer (INL) and in the outer plexiform layer (OPL) but did not extend into the ONL. Based on the pattern of DAF staining, it is unlikely that the DAF positive processes are the same as those positive for GFAP. We therefore performed double labelling of retinal sections from 1 year *Cfh* ^−/−^ mice for DAF (Figure 7A) and the Müller cell marker, glutamine synthetase (Figure 7B). Glutamine synthetase was expressed in Müller cells extending from the GCL to the outer limiting membrane (OLM) however because the primary antibody was raised in mouse, the secondary anti-mouse IgG also stained retinal blood vessels (Figure 7B). A merged image showed that DAF positive cells were also positive for glutamine synthetase (Figure 7C) indicating that DAF is up-regulated in Müller cells. However, DAF positive regions of Müller cells were evident from the GCL to the OPL but did not extend as far as the OLM. High power images of the areas in white boxes show the areas of co-localisation more clearly (Figure 7 D-F).

## Discussion

CFH is one of many complement regulatory genes expressed in the retina [19]. The main site of CFH synthesis within the retina appears to be the RPE [20], from where it may be secreted directly into retina to provide localised protection against unwanted complement activation. Experiments using primary RPE cells in culture revealed enhanced CFH secretion in response to inflammatory stimuli, suggesting that inflammation in AMD may lead to disturbances in retinal complement homeostasis [21]. Various mouse models have been developed that recapitulate some of the features of AMD, such as subretinal deposits, lipofuscin accumulation and choroidal neovascularisation, but thus far none have been established based on the high-risk haplotypes that predispose humans to AMD. One study reported transgenic mice in which mouse-human chimaeric CFH variants were expressed as transgenes against the *Cfh* ^−/−^ background. However, whilst certain AMD-like features developed with time in these animals, there was no difference between the CFH^402H^ and CFH^402Y^ variants [22]. Although the *Cfh* ^−/−^ mouse is not a model for AMD, it does give an insight into the role of complement regulation in the retina that in turn may advance our understanding of how complement dysfunction contributes to the development of AMD.

CFH and other complement genes exhibit changes in their expression levels as the retina ages [12], [23]. Ageing leads to structural and functional changes in all compartments of the retina including the neuroretina, RPE, Bruch’s membrane and ocular perfusion. In this study we observed several features that were consistent with normal ageing in wild-type mice such as accumulation of lipofuscin in the RPE, a decrease in photoreceptor density, a higher propensity for the photoreceptors to detach and loss of polarisation of RPE mitochondria. The majority of these changes also occurred in *Cfh* ^−/−^ mice, with the exception of the loss of polarisation of mitochondria. Indeed, we observed that mitochondria became more polarised with ageing in *Cfh* ^−/−^ mice. The reasons for this are not obvious, but it could be a response to changes in localised energy demands within the RPE or a decrease in height of the basal infoldings.

The young wild-type and *Cfh* ^−/−^ retinas were similar with regard to DAF and GFAP staining, photoreceptor density and visual function. However, as the mice aged we began to observe changes in the *Cfh* ^−/−^ retinas consistent with those previously observed at two years. As in two year old *Cfh* ^−/−^ mice, the photoreceptors at one year showed a greater tendency to detach from the apical processes of the RPE compared to age-matched wild-type controls. Furthermore, thinning of the photoreceptors was enhanced in one year *Cfh* ^−/−^ mice whilst retinal thickness was increased. The former may be due to a combination of neuroretinal stress and RPE dysfunction leading to photoreceptor drop-out whilst the latter may be due to increased blood-retinal barrier permeability or decreased bulk flow of water resulting in mild oedema. In the one year *Cfh* ^−/−^ mice there was no significant difference in the amplitudes of either the a- or b-wave under scotopic conditions from age-matched wild-type controls. This shows that the ERG changes observed in the two year old *Cfh* ^−/−^ mice were a consequence of combined CFH knockout and age. However, we did see an increased time to peak of the scotopic a-wave in one year *Cfh* ^−/−^ mice compared to age-matched wild-type controls. This is perhaps an early indicator of the more extensive loss of visual function seen in older animals.

Immunohistochemical analysis did not reveal any stress-related redistribution of opsins in the photoreceptors (not shown), however we did find early signs of mild retinal stress in astroglia of one year *Cfh* ^−/−^ mice, characterised by GFAP staining. Since glial cells have an intimate relationship with the retinal vasculature, one cause of the glial activation could have been as a reaction to pathological changes to the retinal vasculature. Indeed a study on one year old *Cfh* ^−/−^ mice proposed a role for CFH in ocular perfusion [24]. These investigators reported that the deep plexus of the retinal vessels is withered due to deposition of C3b along endothelial surfaces. However, in this study we observed that the retinal vasculature appeared normal in both young and one year *Cfh* ^−/−^ mice and that there was no sign of C3b deposition on endothelial surfaces (data not shown). It is possible that systemic changes in the mouse due to C3 consumption in the serum lead to retinal stress without causing structural changes in the vasculature. Nevertheless, the presence of both structural and functional changes, although subtle in the one year *Cfh* ^−/−^ mice, indicates that the absence of CFH leads to a mild chronic and progressive retinal pathology.

We recently reported that *Cfh* gene deletion leads to changes in expression of other complement regulators [12], and here we observed significant up-regulation of DAF in Müller cells. Consistent with previous studies in human AMD eyes [19] we observed DAF expression in the GCL in young *Cfh* ^−/−^ and control mouse eyes. DAF has also been identified in human vitreous, where it was proposed to function with membrane cofactor protein and CD59 to control complement activation [25]. Here, the up-regulation of DAF in Müller cells within the inner layers of the retina is consistent with a compensatory response to the loss of CFH, reinforcing the importance of regulating complement activation in the retina.

In summary, we have shown that at a young age there are no significant changes in the retina of the *Cfh* ^−/−^ mouse other than a modest overall thickening. Additionally we show that whilst a number of anatomical and ultrastructural changes are evident by one year in the *Cfh* ^−/−^ mice, many of the more marked structural and functional changes identified in 2 year *Cfh* ^−/−^ mice are not yet present at this time. This highlights the importance of ageing as a predisposing factor in the pathology of *Cfh* ^−/−^ deficiency.

## Materials and Methods

### Animals

*Cfh* ^−/−^ mice were a generously provided by Prof. Matthew Pickering (Imperial College) and have been described previously [10], [11]. Ex-breeding C57Bl/6J control mice of 6–9 months were purchased from Harlan Laboratories, and young C57Bl/6J mice were bred in house in the Biological Resources Unit at the Institute of Ophthalmology. The experiments described in this study were carried out under licence from the UK Home Office and following approval from the UCL Institute of Ophthalmology Ethical Review Panel. Mice were killed with a rising CO_2_ concentration with subsequent cervical dislocation, and eyes were removed immediately. Mice were used at either 7–8 weeks or 1 year ±11 days.

### Electroretinograms and Visual Evoked Potentials

Electroretinogram (ERG) and visual evoked potential (VEP) recordings were performed as described previously [26]. Briefly, mice were dark-adapted overnight and prepared for recordings under dim red illumination. Mice were anaesthetised by intraperitoneal injection with ketamix, 2 µl/g body weight (37.5% ketamine, 25% Dormitor, Fort Dodge Animal Health Ltd) and 10 µl atropine sulphate (600 µg/ml, Hameln Pharmaceuticals). Stimuli for the scotopic recordings were brief full field flashes of white light from darkness at varying intensity (-5.5 to 1 log cd/s/m^2^) by altering both the duration (3 µs-1 ms) and attenuation (1000-1) of the stimulus. The frequency of the flashes was 0.67 Hz at lower intensities, which was decreased to 0.33 and 0.17 Hz at the intermediate and higher intensities. For photopic recordings, mice were light adapted in the recording chamber for 20 min with a rod adapting background light (20 cd). Background light was maintained throughout photopic recordings where brief full field flashes of white light were applied at varying intensity (-1.4 to 1 log cd/s/m^2^) by altering both the duration (1–3 ms) and attenuation (1000-1) of the stimulus. The frequency of flashes was 0.5 Hz.

### Electron Microscopy

For ultrastructural analysis all samples were prepared in the afternoon. For analysis of the RPE, enucleated eyes were immersed in Karnovsky’s fixative (3% glutaraldehyde (EM grade-TAAB, G002), 1% paraformaldehyde in 0.07 M sodium cacodylate (Agar Scientific, R1104), pH 7.4) for 2 h at RT. Sectioning and imaging were performed as described previously [26].

### Organelle Distribution Analysis

RPE cell organelle distribution was analysed from captured transmission electron micrographs of RPE cells in which the nucleus was not visible. Mitochondria were identified by their characteristic internal membraneous stacks, and melanosomes by their uniform electron dense appearance. The distance from the RPE basal lamina to the centre of each organelle was measured using Image J software [27]. More than 500 mitochondria and 900 melanosomes were quantified from five mice in each group.

### Immunostaining

Mouse eyes were fixed in 4% paraformaldehyde, cryopreserved in OCT embedding matrix and cut into 12 µm sections. Sections were blocked and permeabilised in PBS containing 5% normal donkey serum, 1% bovine serum albumin, 0.5% triton X-100 and 0.12% sodium azide. Sections were either stained with a GFAP antibody conjugated to Cy3 fluorophore (Sigma Aldrich, 1∶500 dilution), a DAF antibody (Rat monoclonal antibody, a kind gift from Prof. B. Paul Morgan, University of Cardiff, 1∶50 dilution) or double labeled with glutamine synthetase antibody (Millipore, mouse monoclonal antibody, 1∶200 dilution) all overnight at 4°C. Sections stained with DAF antibody were then washed and stained with anti-rat IgG conjugated to AlexaFluor 488 (1∶200 dilution) for 1 h at RT. Sections dual labeled with glutamine synthetase were also stained with anti-mouse IgG conjugated to AlexaFluor 555 (1∶200 dilution). Sections were again washed and incubated with 1 µg/ml DAPI for 2 min at RT before mounting in Mowial mounting medium.
